# Chloroplast and mitochondrial DNA editing in plants

**DOI:** 10.1038/s41477-021-00943-9

**Published:** 2021-07-01

**Authors:** Beum-Chang Kang, Su-Ji Bae, Seonghyun Lee, Jeong Sun Lee, Annie Kim, Hyunji Lee, Gayoung Baek, Huiyun Seo, Jihun Kim, Jin-Soo Kim

**Affiliations:** 1 Center for Genome Engineering, Institute for Basic Science, Daejeon, Republic of Korea; 2 Department of Chemistry, Seoul National University, Seoul, Republic of Korea

**Keywords:** Molecular engineering in plants, Genetic engineering

## Abstract

Plant organelles including mitochondria and chloroplasts contain their own genomes, which encode many genes essential for respiration and photosynthesis, respectively. Gene editing in plant organelles, an unmet need for plant genetics and biotechnology, has been hampered by the lack of appropriate tools for targeting DNA in these organelles. In this study, we developed a Golden Gate cloning system^1^, composed of 16 expression plasmids (8 for the delivery of the resulting protein to mitochondria and the other 8 for delivery to chloroplasts) and 424 transcription activator-like effector subarray plasmids, to assemble DddA-derived cytosine base editor (DdCBE)^2^ plasmids and used the resulting DdCBEs to efficiently promote point mutagenesis in mitochondria and chloroplasts. Our DdCBEs induced base editing in lettuce or rapeseed calli at frequencies of up to 25% (mitochondria) and 38% (chloroplasts). We also showed DNA-free base editing in chloroplasts by delivering DdCBE mRNA to lettuce protoplasts to avoid off-target mutations caused by DdCBE-encoding plasmids. Furthermore, we generated lettuce calli and plantlets with edit frequencies of up to 99%, which were resistant to streptomycin or spectinomycin, by introducing a point mutation in the chloroplast 16S rRNA gene.

## Abstract (web summary)

This study showed efficient base editing of mitochondria and chloroplast genomes in both lettuce and rapeseed by DddA-derived cytosine base editors (DdCBEs), and DNA-free editing in chloroplasts by delivering DdCBE mRNA to protoplasts, which avoids off-target effects.

## Main

Programmable genome editing tools, which include zinc-finger nucleases^3^, transcription activator-like effector (TALE) nucleases^4^, clustered regularly interspaced short palindromic repeat (CRISPR) systems^5–8^ and base editors^9–11^ composed of the catalytically deficient CRISPR-associated protein 9 (Cas9) variant and a nucleobase deaminase protein, have been developed for plant genetic studies and crop improvements through the manipulation of genomic DNA sequences. However, these tools come short of editing DNA sequences in plant organelles, including mitochondria and chloroplasts, possibly because it is difficult to deliver both guide RNA and the Cas9 protein to organelles or to express the two components in organelles simultaneously. Plant organelle genomes encode many genes essential for photosynthesis and respiration. Methods or tools for editing these genes in organelles are highly desired for studying the functions of these genes and improving crop productivity and traits. For example, targeted mutagenesis in the mitochondria *atp6* gene can give rise to male sterility^12^, a useful trait for breeding, whereas a specific point mutation in the 16S rRNA gene in the chloroplast genome leads to antibiotic resistance^13^, as shown below.

Recently, Mok et al.^2^ demonstrated that CRISPR-free DddA-derived cytosine base editors (DdCBEs) enable targeted C∙G-to-T∙A base substitutions in mitochondrial DNA in mammalian cells. DdCBEs composed of non-toxic split domains of the bacterial cytidine deaminase toxin (DddA_tox_), a custom-designed TALE array and a uracil glycosylase inhibitor (UGI) function as heterodimers to catalyse cytosine deamination, inducing C-to-T conversions, within a spacer region between the two TALE protein binding sites in target DNA. In this study, we present a rapid and convenient system to assemble DdCBE plasmids for expression in mitochondria and chloroplasts and use the resulting DdCBEs to demonstrate highly efficient organelle base editing in plants (Supplementary Fig. 1).

**Fig. 1:**
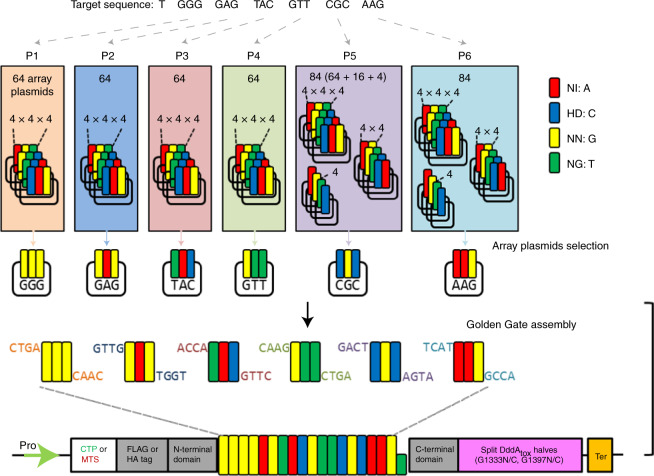
Golden Gate assembly system of plant organelle base editors. Schematic illustration of Golden Gate assembly for cp-DdCBE and mt-DdCBE construction. For each position in a target sequence, TALE subarray plasmids were selected from a total set of 424 (6 × 64 tripartite + 2 × 16 bipartite + 2 × 4 monopartite) and mixed with a destination vector to generate plasmids encoding DdCBEs targeted to specific sequences.

To this end, we first developed a Golden Gate assembly system to construct chloroplast-targeting DdCBE (cp-DdCBE) plasmids or mitochondrial-targeting DdCBE (mt-DdCBE) plasmids (Fig. 1). Our expression plasmids encode fusion proteins composed of a chloroplast transit peptide (CTP) or a mitochondrial targeting sequence (MTS), the TALE N- or C-terminal domains, split-DddA_tox_ halves (G1333N, G1333C, G1397N and G1397C) and UGI, which are codon-optimized for expression in dicot plants, under the control of the parsley ubiquitin (PcUbi) promoter and pea3A terminator. DdCBE plasmids with custom-designed TALE DNA-binding arrays can be constructed in a single subcloning step by mixing an expression vector and six TALE subarray plasmids in an Eppendorf tube. A total of 424 (6 × 64 tripartite + 2 × 16 bipartite + 2 × 4 monopartite) modular TALE subarray plasmids^1^ are available for making cp-DdCBEs or mt-DdCBEs that recognize DNA sequences of 16–20 base pairs in length, including a conserved T at the 5′ terminus. As a result, a functional DdCBE heterodimer recognizes 32- to 40-base-pair DNA sequences.

**Fig. 2:**
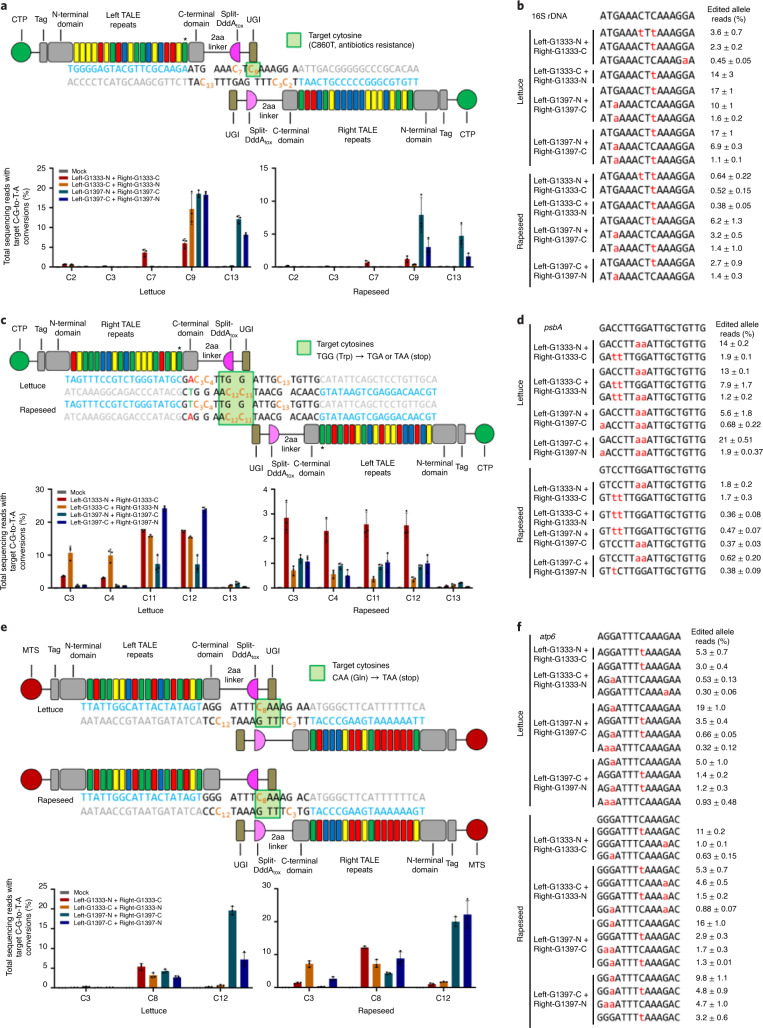
Chloroplast and mitochondrial base editing in plants. **a**–**d**, Frequencies and patterns of chloroplast base editing induced by cp-DdCBE in 16S rDNA (**a**,**b**) and *psbA* (**c**,**d**). Split DdCBE G1333 and G1397 pairs were transfected into lettuce and rapeseed protoplasts. **e**,**f**, Editing efficiencies and patterns of mitochondrial base editing induced by mt-DdCBE in the *atp6* gene. Split DdCBE G1333 and G1397 pairs were transfected into lettuce and rapeseed protoplasts. In **a**,**c**,**e**, the TALE-binding regions are shown in blue, and the cytosines in the spacer are shown in orange. In all graphs, the error bars indicate the mean ± s.d. of three independent biological replicates. For **a** and **c**, the last TALE repeat (*) does not match the reference sequence. In **b**,**d**,**f**, converted nucleotides are shown in red. Edited allele percentages (mean ± s.d.) were obtained from three independent experiments.

To assess whether our DdCBEs can promote base editing in chloroplasts, we constructed four pairs of cp-DdCBE plasmids specific to the chloroplast 16S rRNA gene encoding the RNA component of the 30S ribosomal subunit, co-transfected each pair into lettuce and rapeseed protoplasts, and measured base editing efficiencies using targeted deep sequencing at day 7 post-transfection (Fig. 2a,b). The best-performing cp-DdCBE pair (Left-G1397-N + Right-G1397-C) induced C∙G-to-T∙A conversions in the 15-base-pair spacer region between the two TALE array-binding sites at frequencies of 30% in lettuce protoplasts and 15% in rapeseed protoplasts (Fig. 2b). In line with the previous results in mammalian cells^2^ and mice^14^, cytosines (C9 and C13) in a 5′-TC motif were converted to thymine preferentially by this cp-DdCBE. Interestingly, a cytosine (C7) in a 5′-AC context was changed to thymine at a frequency of 4.2% in lettuce protoplasts by another cp-DdCBE (Left-G1333-N + Right-G1333-C). We also investigated the persistence of cp-DdCBE-mediated base editing in lettuce protoplasts over 14 days of cultivation (Supplementary Fig 2). Editing efficiencies continuously increased for up to 10 days and were maintained throughout the period of cultivation.

We also tested base editing in two additional chloroplast genes, *psbA* and *psbB*, which encode the photosynthetic proteins, D1 and CP-47, respectively, of Photosystem II (Fig. 2c,d and Supplementary Fig. 3). Among four cp-DdCBEs targeted to the *psbA* gene, the most active one (Left-G1397-C + Right-G1397-N) was able to induce C∙G-to-T∙A conversions in lettuce protoplasts with frequencies of up to 25% (Fig. 2d). Only the two cytosines (C11 and C12) in a 5′-TCC context were efficiently converted to thymines by this base editor. It is possible that 5′-TCC was first converted to 5′-TTC and then to 5′-TTT. In rapeseed protoplasts, the other split pair (Left-G1333-N + Right-G1333-C) was most active at four cytosine positions (C3, C4, C11 and C12) with editing efficiencies of up to 3.5% (C3). Note that C3 and C4 are in a 5′-TCC context in the rapeseed gene, whereas they are in a 5′-ACC context in the lettuce counterpart, owing to a single nucleotide polymorphism, which is responsible for efficient editing of the two cytosines (C3 and C4) in the rapeseed gene but not in the lettuce gene by this DdCBE. Likewise, the cp-DdCBE pair targeted to the *psbB* gene catalysed the conversion of two cytosines in a TCC context at editing frequencies of 0.36% to 4.1% in rapeseed protoplasts (Supplementary Fig. 3). Taken together, these results suggest that editing efficiencies depend on cytosine positions and contexts within a spacer region as well as DddA_tox_ split positions (G1333 versus G1397) and orientations (Left-G1333-N versus Left-G1333-C) and demonstrate that our cp-DdCBEs enable efficient base editing in the chloroplast genome in plants.

Next, we sought to achieve base editing in plant mitochondrial DNA using our custom-designed mt-DdCBEs. To this end, we constructed mt-DdCBE-encoding plasmids (using our Golden Gate cloning system) targeted to the *atp6* gene in lettuce and rapeseed and the *rps14* gene in rapeseed, transfected the resulting plasmids into lettuce and rapeseed protoplasts, and measured base editing frequencies using targeted deep sequencing at day 7 post-transfection (Fig. 2e,f and Supplementary Fig. 4). The most active mt-DdCBE pairs (Left-G1397-N + Right-G1397-C in lettuce and Left-G1397-C + Right-G1397-N in rapeseed) were able to catalyse C∙G-to-T∙A conversions at the *atp6* target site with a frequency of 23% in lettuce protoplasts and 23% in rapeseed protoplasts (Fig. 2f). Also, the mt-DdCBE pair induced C∙G-to-T∙A conversions at the *rps14* target site with frequencies of 11% in rapeseed protoplasts (Supplementary Fig. 4). These results show that mitochondrial DNA in plants is amenable to base editing with mt-DdCBEs.

**Fig. 3:**
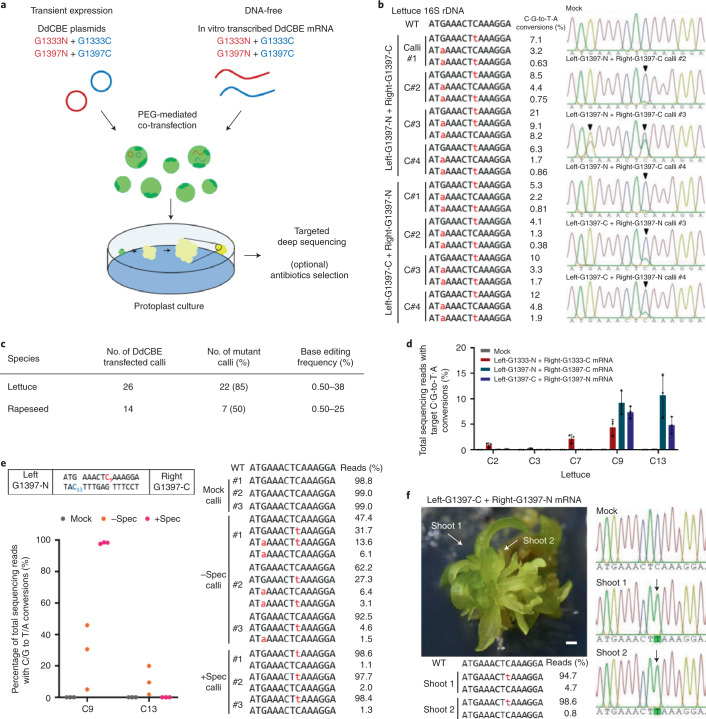
Plant organelle DNA editing via DdCBEs. **a**, Schematic diagram of plant organelle mutagenesis. **b**, The efficiencies of C∙G-to-T∙A conversions in cp-DdCBE-transfected calli cultured in the absence of spectinomycin, with representative Sanger sequencing chromatograms. Converted nucleotides are shown in red in the sequences on the left. The arrowheads indicate the substituted nucleotides in the chromatograms. WT, wild type. **c**, Summary of DdCBE-driven plant organelle mutagenesis. Mutant calli are defined as those with edit frequencies significantly higher than the frequencies in mock-treated calli. **d**, Frequencies of C-to-T conversions induced following the transfection of mRNA encoding cp-DdCBE targeted to 16S rDNA into lettuce protoplasts. The error bars are the mean ± s.d. of *n* = 3 independent biological replicates. **e**, Frequencies and editing patterns in 2.5-month-old, spectinomycin-resistant calli. Spec, spectinomycin. **f**, Efficiencies of C∙G-to-T∙A conversions in DdCBE mRNA-transfected, streptomycin-resistant plantlets, with representative Sanger sequencing chromatograms. The arrows indicate the substitute nucleotides in the chromatograms. Scale bar, 1 mm.

To investigate whether DdCBE-mediated edits in cpDNA and mtDNA were maintained during regeneration, we collected lettuce and rapeseed calli regenerated from DdCBE-treated protoplasts, four weeks after transfection (Fig. 3a), and measured base editing efficiencies in each callus using targeted deep sequencing and Sanger sequencing (Fig. 3b and Supplementary Fig. 5). Base edits induced by the DdCBE specific to the chloroplast or mitochondrial genes were detected in 22 out of 26 lettuce calli and 7 out of 14 rapeseed calli with frequencies of up to 38% and 25%, respectively (Fig. 3c). Also, base edits in the chloroplast *psbA* gene were observed with frequencies of up to 3.9% in lettuce calli (Supplementary Fig. 5). Likewise, mitochondrial base edits were detected in rapeseed calli with frequencies of up to 25% and 1.9% in the *atp6* and *rps14* target sites, respectively (Supplementary Fig. 5). These results show that DdCBE expression in plant protoplasts can be tolerated and that organelle base edits induced by DdCBEs in protoplasts remain intact during regeneration.

We then sought to demonstrate DNA-free base editing in organelles using in vitro transcribed cp-DdCBE mRNA rather than expression plasmids. We transfected in vitro transcripts encoding the cp-DdCBE targeted to the 16S rRNA gene into lettuce protoplasts and analysed base editing frequencies at the target site (Fig. 3a). C-to-T mutations were detected in protoplasts with frequencies of up to 25% (Fig. 3d and Supplementary Fig. 6). As expected, DdCBE mRNA or DNA sequences were absent in protoplasts at day 7 post-transfection (Supplementary Fig. 7). This method can avoid potential integration of plasmid DNA fragments in the host genome.

Encouraged by the stable maintenance of organelle edits in calli regenerated from protoplasts, we investigated whether the chloroplast DNA edits in the 16S rRNA gene could confer resistance to streptomycin and spectinomycin, antibiotics that bind to 16S rRNA irreversibly, leading to the inhibition of protein synthesis. Several single nucleotide polymorphisms in the 16S rRNA gene are commonly observed in streptomycin-resistant prokaryotes and eukaryotes; in particular, the 16S rRNA C860T (*Escherichia coli* coordinate C912) mutation endows *Nicotiana tabacum* (tobacco) with resistance to streptomycin^13^. The nucleotide affected by the C860T point mutation in tobacco and the equivalent nucleotide in lettuce correspond to the C9 position in the lettuce gene (Figs. 2a,b and 3b,d). We transferred lettuce calli regenerated from DdCBE-treated protoplasts to medium containing streptomycin or spectinomycin. Mock-treated calli turned white, indicative of protoplast dysfunction, upon exposure to antibiotics (Supplementary Fig. 8). In contrast, DdCBE-treated calli remained greenish, showing resistance to these antibiotics. We analysed DdCBE editing efficiencies in the resulting antibiotic-resistant lettuce calli and plantlets. C-to-T conversions at the C9 position, corresponding to the C860T mutation, were observed at high frequencies of up to 98.6% in calli and shoots regenerated from the drug-resistant calli (Fig. 3e,f). Interestingly, bystander C-to-T edits at the nearby C13 position were detected at frequencies of up to 20% in the absence of spectinomycin but not at all in the presence of the antibiotic, demonstrating selection against this mutation upon drug treatment. Taken together, these results show that plant organelle mutations induced by DdCBEs in protoplasts can be maintained after cell division and plant development and that near homoplasy of chloroplast edits can be achieved by drug selection.

**Fig. 4:**
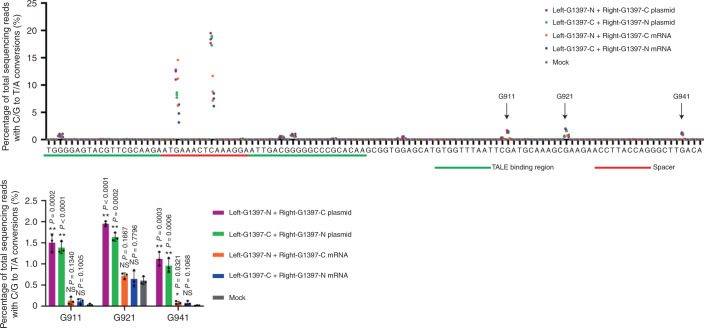
Comparison of off-target activity in the vicinity of the target site in DdCBE plasmid-transfected or DdCBE mRNA-transfected lettuce protoplasts. Plasmids or mRNAs encoding the cp-DdCBE pair targeted to the chloroplast 16S rRNA gene were transfected into lettuce protoplasts. Off-target TC-to-TT edits were detected in the immediate proximity of the target site. Editing efficiencies were measured by targeted deep sequencing seven days post-transfection. Frequencies (mean ± s.d.) were obtained from three independent experiments. Student’s unpaired two-tailed *t*-test was applied. **P < 0.01; *P < 0.05; NS, not significant (*P* > 0.05).

We also analysed the off-target activity of the TALE deaminase targeted to the 16S rRNA site in protoplasts, calli and shoots. No off-target mutations were detectably induced in antibiotic-resistant calli or shoots, which were derived from single cells, in the vicinity (±50 base pairs) of the target site (Supplementary Fig. 9) or at the top five candidate off-target sites in the chloroplast genome, which were chosen on the basis of sequence homology (Supplementary Fig. 10). In contrast, when plasmids encoding the DdCBE pair were used to transfect protoplasts, off-target TC-to-TT mutations were induced in the proximity of the target site and at three of the five candidate off-target sites with low frequencies that ranged from 1.2% to 4.1% (Supplementary Fig. 10). The use of in vitro transcripts (mRNA) instead of plasmids encoding the TALE deaminase largely avoided these off-target activities in protoplasts (Fig. 4). These results suggest that overexpression or prolonged, plasmid-based expression of DdCBEs can give rise to off-target mutations and that transient, mRNA-based expression using mRNA is desirable for avoiding off-target base editing.

In summary, we have developed a Golden Gate cloning system, which employs a total of 424 TALE subarray plasmids and 16 expression plasmids, to assemble DdCBE-encoding plasmids for organelle base editing in plants. Our DdCBEs custom-designed to target three genes in chloroplast DNA and two genes in mitochondrial DNA achieved C-to-T conversions at high frequencies in lettuce and rapeseed protoplasts. Importantly, the edits in plant organelles were maintained during cell division and plant development. Furthermore, we were able to obtain antibiotic-resistant lettuce calli and plantlets with near homoplasy (99%) by inducing a mutation in the chloroplast 16S rRNA gene. Even without antibiotic selection, edit frequencies were as high as 25% in mitochondria and 38% in chloroplasts. Further studies are warranted to investigate whether DdCBE-induced heteroplasy gives rise to phenotypic effects and whether organelle editing efficiencies can be enhanced by engineering DdCBEs. We expect that our Golden Gate cloning system will be a valuable resource for organelle DNA editing in plants.

## Methods

### Construction of plasmids for expression in plant protoplasts

DdCBE Golden Gate destination vectors were constructed using Gibson assembly (New England Biolabs). Sequences encoding the TAL N-terminal domain, HA tag, FLAG tag, TAL C-terminal domain, split-DddA_tox_ and UGI were codon-optimized for expression in dicot (*Arabidopsis thaliana*) plants and synthesized by Integrated DNA Technology. The sequence encoding the CTP from *AtinfA*^15^ and AtRbcS^16^ and the MTS from the ATPase delta subunit^17^ and ATPase gamma subunit^18^ were amplified from *A. thaliana* cDNAs. The PcUbi promoter and pea3A terminator were used to replace the mammalian CMV promoter in a backbone plasmid for plant expression^1^. To construct the vector for in vitro DdCBE mRNA transcription, a T7 promoter cassette was cloned into the DdCBE Golden Gate destination vector between the PcUbi promoter and the DdCBE coding region.

TALE array genes were cloned by one-way Golden Gate assembly^1^. Plasmids expressing DdCBE were constructed by BsaI digestion and T4 DNA ligation of Golden Gate assembly products using 424 TALE array plasmids and destination vectors. One-way Golden Gate cloning was performed using the following steps: 20 cycles of 37 °C and 50 °C for 5 min each, followed by final incubations at 50 °C for 15 min and 80 °C for 5 min. All vectors for plant protoplast transfection were purified using Plasmid Plus Midiprep kits (Qiagen). The DNA and amino acid sequences used in vector construction are provided in Supplementary Sequences 1–3.

### mRNA in vitro transcription

DdCBE DNA templates were prepared by PCR using Phusion High-Fidelity DNA Polymerase (Thermo Scientific). DdCBE mRNAs were synthesized and purified using an in vitro mRNA synthesis kit (Enzynomics). The primers for DNA template PCR amplification are listed in Supplementary Table 1.

### Protoplast isolation and transfection

Lettuce (*Lactuca sativa* cv. Cheongchima) seeds were surface sterilized in 70% ethanol for 30 s and in a 0.4% hypochlorite solution for 15 min and were washed three times in distilled water. The lettuce seeds were germinated on 0.5× Murashige and Skoog (MS) medium supplemented with 2% sucrose under conditions of 16 h light and 8 h dark at 25 °C. Rapeseed (*Brassica napus* cv. Halla) seeds were surface sterilized in 70% ethanol for 3 min and in a 1.0% hypochlorite solution for 30 min, after which they were washed three times with distilled water. The rapeseed seeds were germinated on 1× MS medium supplemented with 3% sucrose under conditions of 16 h light and 8 h dark at 23 °C.

Protoplast isolation and transfection were performed as described previously^8,11^. Cotyledons from 7-day-old lettuce and 14-day-old rapeseed plants were digested with enzyme solution (1% viscozyme, 0.5% celluclast, 0.5% novozyme, 3 mM MES, 9% mannitol and CPW salts, pH 5.8) during incubation with shaking (40 r.p.m.) in the dark at room temperature for 3 h. The protoplast–enzyme mixture was washed with an equal volume of W5 solution (154 mM NaCl, 125 mM CaCl_2_, 5 mM KCl, 5 mM glucose and 1.5 mM MES, pH 5.6), and intact protoplasts were harvested on a sucrose gradient (21%) by swing-out centrifugation at 80 *g* for 7 min. The protoplasts were incubated in W5 solution for 1 h at 4 °C before polyethylene glycol (PEG)-mediated transfection.

Lettuce protoplasts (5 × 10^5^) and rapeseed protoplasts (2 × 10^5^) resuspended in MMG solution (0.4 M mannitol, 15 mM MgCl_2_ and 4 mM MES, pH 5.7) were transfected with plasmids (30 μg per construct) or mRNAs (40 μg per transcript) by PEG (40% (w/v) PEG 4,000, 0.2 M mannitol and 0.1 M CaCl_2_)-mediated transfection and incubated for 20 min at room temperature. The PEG–protoplast mixture was washed three times with an equal volume of W5 solution with gentle inverting and incubated for 10 min. The protoplasts were then pelleted by swing-out centrifugation at 100 *g* for 5 min.

### Protoplast culture

Lettuce protoplasts transfected with DdCBE-encoding plasmids were resuspended in lettuce protoplast culture medium^19^ (LPCM) (0.5× B5 culture medium supplemented with 375 mg l^−1^ CaCl_2_ ∙ 2H_2_O, 18.35 mg l^−1^ NaFe-EDTA, 270 mg l^−1^ sodium succinate, 103 g l^−1^ sucrose, 0.2 mg l^−1^ 2,4-dichlorophenoxyacetic acid (2,4-D), 0.3 mg l^−1^ 6-benzylaminopurine (BAP) and 0.1 g l^−1^ MES). The protoplasts in LPCM were mixed 1:1 with LPCM containing 2.4% low-melting-point agarose (agarose type VII) and immediately plated on a six-well plate. After the mixture solidified, the embedded protoplasts were overlaid with 1 ml of LPCM and incubated at 25 °C for one week in the dark. After this initial incubation, the overlaid LPCM was replaced with fresh LPCM every week, and the embedded protoplasts were incubated at 25 °C under conditions of 16 h dim light and 8 h dark for one week and then of 16 h light and 8 h dark for two weeks. Micro-calli induced from protoplasts were cultured on regeneration medium (1× MS regeneration medium supplemented with 30 g l^−1^ sucrose, 0.1 mg l^−1^ α-naphthaleneacetic acid (NAA), 0.5 mg l^−1^ BAP and 0.7% plant agar) for four weeks under conditions of 16 h light and 8 h dark at 25 °C. In preparation for analysis of the base editing efficiency, transfected protoplasts were cultured in LPCM at 25 °C in the dark for one week without embedding. To examine antibiotic resistance, one-month-old embedded micro-calli were transferred on regeneration medium containing 50 mg l^−1^ streptomycin or 50 mg l^−1^ spectinomycin for four weeks under conditions of 16 h light and 8 h dark at 25 °C. After four weeks, antibiotic-resistant green calli or adventitious shoots were transferred to a fresh regeneration medium containing 200 mg l^−1^ streptomycin or 50 mg l^−1^ spectinomycin.

Rapeseed protoplasts transfected with DdCBE-encoding plasmids were resuspended in rapeseed protoplast culture medium^20^ (RPCM) (1× B5 culture medium supplemented with 0.6 g l^−1^ CaCl_2_, 20 g l^−1^ glucose, 70 g l^−1^ mannitol, 1 mg l^−1^ NAA, 1 mg l^−1^ BAP and 0.25 mg l^−1^ 2.4-D). The protoplast–RPCM mixture was transferred into a six-well plate and incubated at 25 °C for two weeks in the dark. After two weeks, the protoplasts were incubated at 25 °C under conditions of 16 h dim light and 8 h dark for three weeks. The RPCM was replaced with fresh RPCM every week.

### DNA and RNA extraction

Total DNAs or RNAs were extracted from cultured cells in liquid medium and transgenic calli using a DNeasy Plant Mini Kit or RNeasy Plant Mini Kit (Qiagen). Cultured cells and calli were harvested by centrifugation at 10,000 r.p.m. for 1 min. The cDNA from total RNA was reverse-transcribed using RNA to cDNA EcoDry Premix (Oligo dT) (Takara).

### Targeted deep sequencing

Target regions were amplified using Phusion High-Fidelity DNA Polymerase with the appropriate primers (Supplementary Table 1). Three rounds of PCR were performed (first, nested PCR; second, PCR; and third, indexing PCR) to make a DNA sequencing library. Equal amounts of the DNA libraries were pooled and sequenced using MiniSeq (Illumina). The paired-end sequencing files were analysed by the Cas-analyzer (http://www.rgenome.net)^21^ and source code of the computer program at https://github.com/ibs-cge/maund.

### Reporting Summary

Further information on research design is available in the Nature Research Reporting Summary linked to this article.

## Supplementary information

Supplementary Information Supplementary Figs. 1–10, Table 1 and Sequences 1–3.

Reporting Summary

Supplementary Data 1 Unprocessed gels for Supplementary Fig. 7.

## Data Availability

The data that support the findings of this study are available from the corresponding author upon request. The high-throughput sequencing data from this study have been deposited in the NCBI BioProject database under the accession codes PRJNA727868 and PRJNA727869.

## Code availability

The source code used to calculate the substitution frequencies can be accessed at https://github.com/ibs-cge/maund.
