# Supplementary material for: Chloroplast and mitochondrial DNA editing in plants
Source: Nat Plants. 2021 Jul 1;7(7):899–905. doi: 10.1038/s41477-021-00943-9 (PMC8289734; doi:10.1038/s41477-021-00943-9)
Supplement: Supplementary file 1 — Supplementary Figs. 1–10, Table 1 and Sequences 1–3. [file 41477_2021_943_MOESM1_ESM.pdf]

nature portfolio

https://doi.org/10.1038/s41477-021-00943-9

Supplementary information

Chloroplast and mitochondrial DNA editing in plants

In the format provided by the authors and unedited

Supplementary Information

Chloroplast and mitochondrial DNA editing in plants

Table of Contents

Supplementary Figure

Supplementary Figure 1. Chloroplast and mitochondrial base editing strategy.

Supplementary Figure 2. Time course of DdCBE plasmid-mediated editing in lettuce protoplasts.

Supplementary Figure 3. Frequencies of base editing in the *psbB* gene.

Supplementary Figure 4. Base editing efficiencies in the mitochondrial *rps14* gene.

Supplementary Figure 5. Chloroplast and mitochondrial genome targeted base editing efficiencies in calli.

Supplementary Figure 6. DNA-free base editing.

Supplementary Figure 7. PCR and RT-PCR of DdCBE sequences in protoplasts and calli.

Supplementary Figure 8. Selection of 16s rDNA mutants.

Supplementary Figure 9. No off-target mutations were detected in the immediate proximity of the DdCBE target site in antibiotic-resistant calli or plantlets.

Supplementary Figure 10. Analysis of off-target activity at the 5 sites with the most homology with the on-target site.

Supplementary Table

Supplementary Table 1. PCR primers used in this study.

Supplementary Sequences

Supplementary Sequences 1. DNA sequences of plasmid components.

Supplementary Sequences 2. Amino acid sequences of DdCBE components.

Supplementary Sequences 3. Amino acid sequences of TALE repeats.

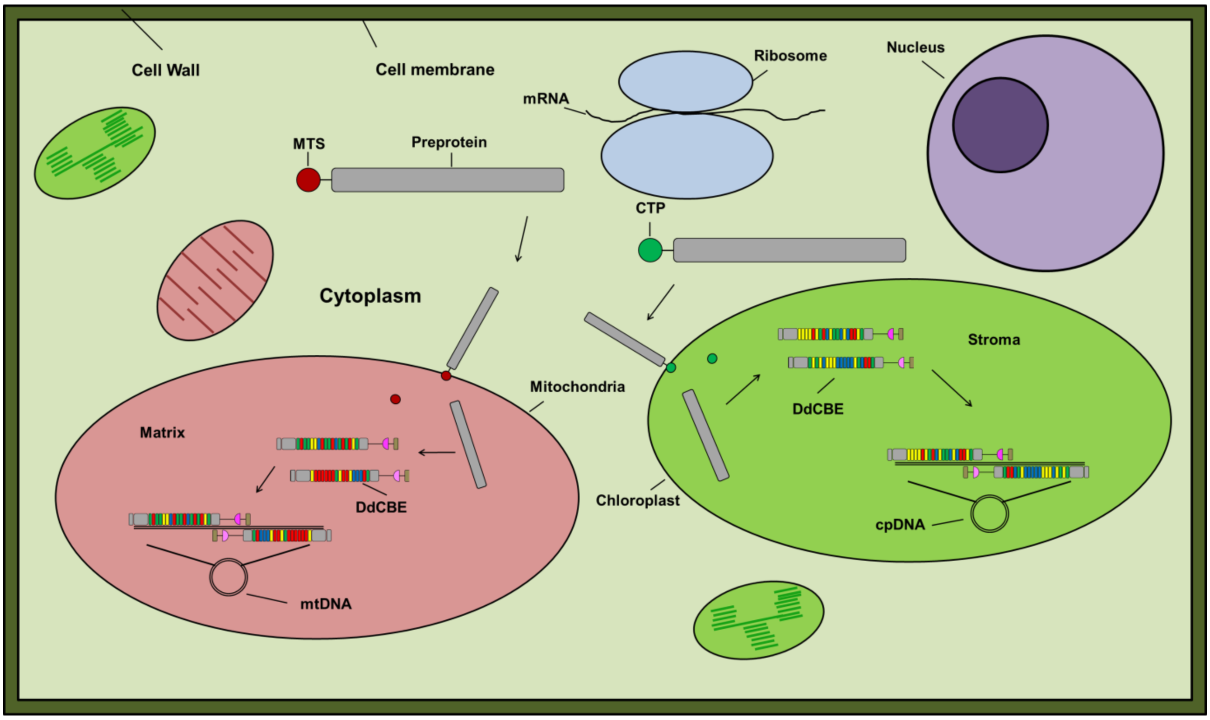

Supplementary Figure 1. Chloroplast and mitochondrial base editing strategy. Because cp-DdCBE and mt-DdCBE preproteins (precursor proteins) respectively contain a chloroplast transit peptide (CTP) or a mitochondrial targeting signal (MTS), they are imported into chloroplasts and mitochondria after translation in plant cells. The preproteins cross the outer and inner organelle membranes, after which CTP and MTS are respectively cleaved by stromal processing peptidase and mitochondrial processing peptidase, and cp-DdCBE and mt-DdCBE (the mature proteins) take on their final conformation.

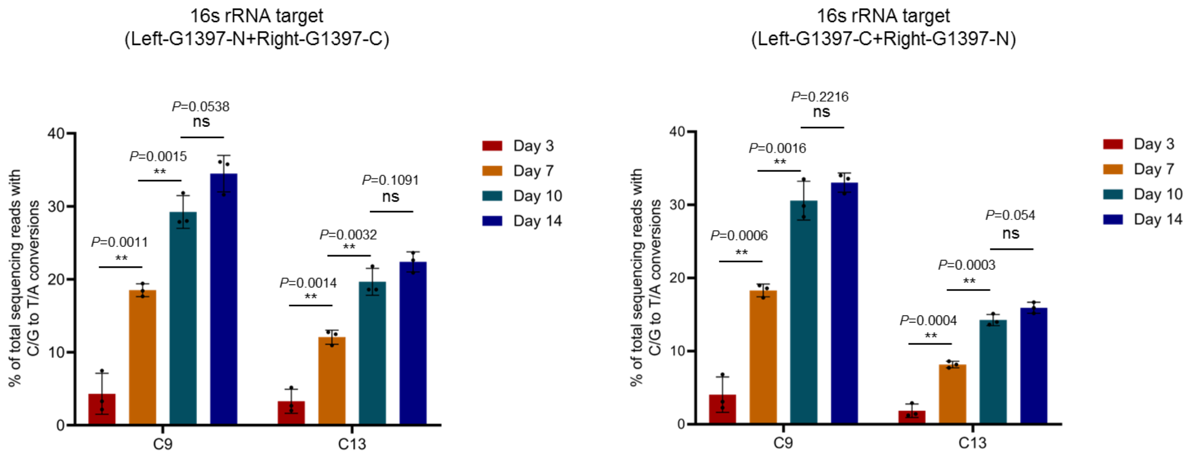

Supplementary Figure 2. Time course of DdCBE plasmid-mediated editing in lettuce protoplasts. Transfected protoplasts were harvested at each time point and analyzed for editing efficiencies by targeted deep sequencing. Frequencies (mean±s.d.) were obtained from three independent experiments. Student's unpaired two-tailed t-test was applied. ***P*<0.01; ns, not significant (P>0.05).

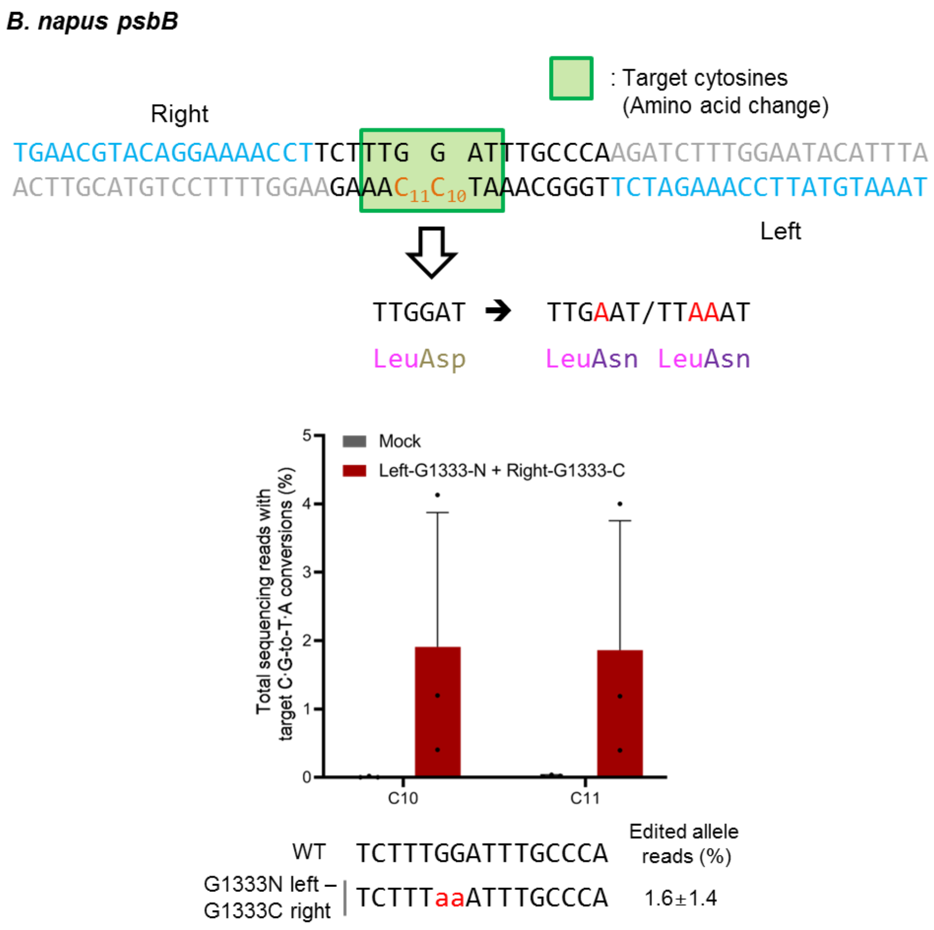

Supplementary Figure 3. Frequencies of base editing in the *psbB* gene. Plasmids encoding the cp-DdCBE pair Left-G1333-N + Right-G1333-C, which is targeted to the chloroplast psbB gene, were transfected into rapeseed protoplasts, after which base editing efficiencies in the spacer region were analyzed by targeted deep sequencing. TALE-binding regions, target cytosines, and converted nucleotides are shown in blue, orange, and red, respectively. Frequencies (mean±s.d.) were calculated from n=3 independent experiments.

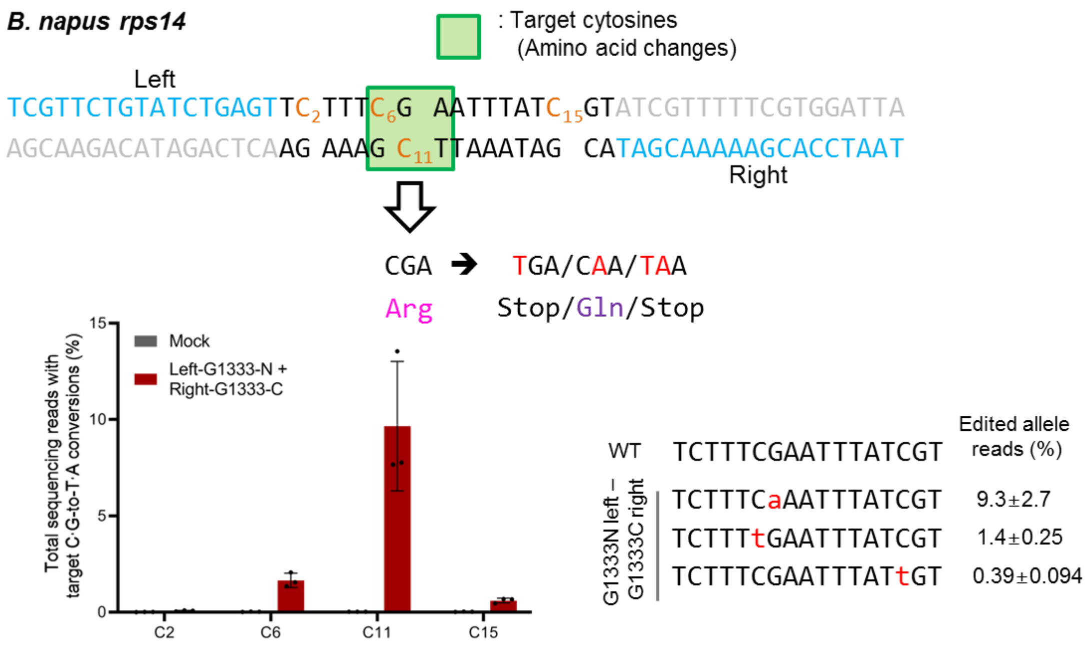

Supplementary Figure 4. Base editing efficiencies in the mitochondrial *rps14* gene. Plasmids encoding the mt-DdCBE pair Left-G1333-N + Right-G1333-C, which is targeted to the RPS14 gene, were transfected into rapeseed protoplasts, after which the efficiencies of C-to-T conversion were analyzed by targeted deep sequencing. TALE binding regions, target cytosines, and converted nucleotides are shown in blue, orange, and red, respectively. Frequencies (mean±s.d.) were calculated from n=3 independent experiments.

(a)

Lettuce 16s rDNA

Sample		Sequence	C·G-to-T·A conversions (%)
WT		ATGAAACTCAAAGGA	
G1333N-Left + G1333C-Right	Callus #1	ATGAAACTtAAAGGA	0.82 %
		ATGAAAtTtAAAGGA	0.67 %
		ATGAAACTCAAAGaA	0.33 %
	C#2	ATGAAACTtAAAGGA	1.02 %
		ATGAAAtTtAAAGGA	0.62 %
	C#3	ATGAAACTtAAAGGA	0.66 %
		ATGAAAtTtAAAGGA	0.33 %
	C#4	ATGAAACTtAAAGGA	0.35 %
		ATGAAAtTtAAAGGA	0.19 %

Rapeseed 16s rDNA

Sample		Sequence	C·G-to-T·A conversions (%)
WT		ATGAAACTCAAAGGA	
G1333N-Left + G1333C-Right	C#1	ATGAAAtTtAAAGGA	0.32 %
		ATGAAACTCAAAaaA	0.25 %
		ATaAAAtTtAAAaaA	0.11 %
		ATGAAAtTtAAAGaA	0.10 %
	C#2	ATGAAAtTtAAAGGA	0.84 %
		ATGAAACTCAAAaaA	0.28 %
		ATGAAACTtAAAaaA	0.10 %

Lettuce *psbA*

Sample		Sequence	C·G-to-T·A conversions (%)
WT		GACCTTGGATTGCTGTTG	
G1333N-Left + G1333C-Right	C#1	GACCTTaaATTGCTGTTG	2.91 %
		GAttTTGGATTGCTGTTG	0.26 %
		GAttTTaaATTGCTGTTG	0.22 %
	C#2	GACCTTaaATTGCTGTTG	3.11 %
		GAttTTaaATTGCTGTTG	0.51 %
		aACCTTaaATTGCTGTTG	0.29 %
	C#3	GACCTTaaATTGCTGTTG	1.36 %
		GAtCTTGGATTGCTGTTG	0.45 %
		GACCTTGGATTaCTGTTG	0.25 %
	C#4	GACCTTaaATTGCTGTTG	2.31 %
		GACCTTGaATTGCTGTTG	0.27 %
		GAttTTGGATTGCTGTTG	0.24 %

(b)

Rapeseed *atp6*

Sample		Sequence	C·G-to-T·A conversions (%)
WT		GGGATTTCAAAGAC	
G1333N-Left + G1333C-Right	C#1	GGGATTTtAAAGAC	24.92 %
	C#2	GGGATTTtAAAGAC	0.41 %
		GGGATTTtAAAaAC	0.13 %

Rapeseed *rps14*

Sample		Sequence	
WT		TCTTTCGAATTTATCGT	
G1333N-Left + G1333C-Right	C#1	TCTTTCaAATTTATCGT	0.52 %
		TCTTTtGAATTTATCGT	0.30 %
	C#2	TCTTTCaAATTTATCaT	1.00 %
		TCTTTCaAATTTATCGT	0.53 %
		TCTTTCGAATTTATCaT	0.37 %

Supplementary Figure 5. Chloroplast and mitochondrial genome targeted base editing efficiencies in calli. (a) The frequencies and patterns of DdCBE-mediated base editing at targeted sites in 16s rDNA and *psbA* in lettuce and rapeseed calli after 4 weeks of culture. (b) The frequencies and patterns of DdCBE-mediated base editing at targeted sites in the *atp6* and *rps14* genes in rapeseed calli, revealed by targeted deep sequencing. Converted nucleotides in the spacer region are shown in red.

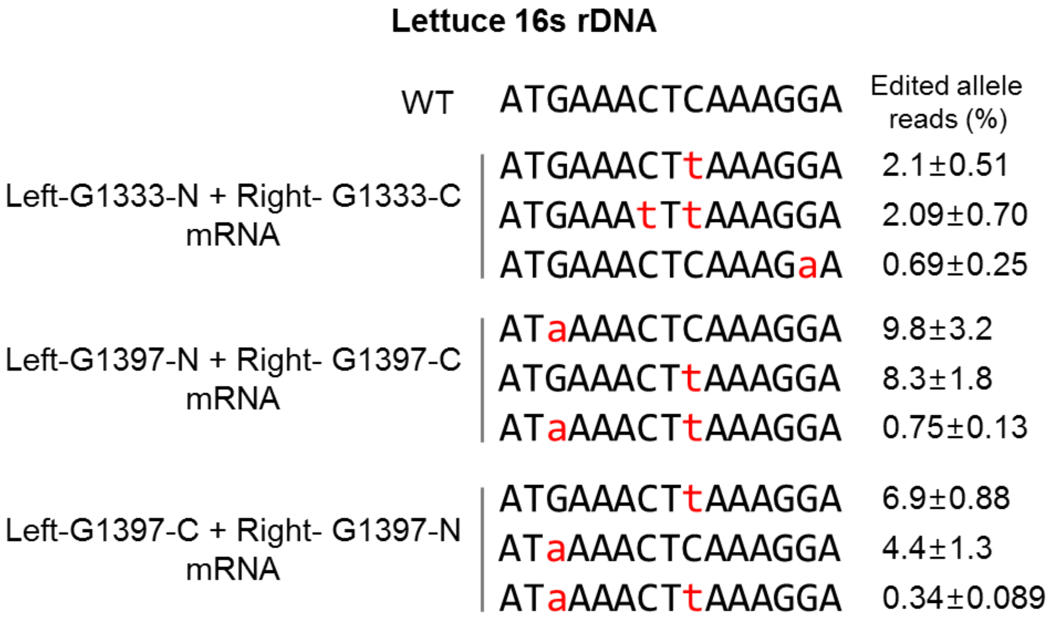

Supplementary Figure 6. DNA-free base editing. Frequencies and patterns of base editing in the chloroplast at the targeted site in 16s rDNA after transfection of DdCBE mRNAs into lettuce protoplasts. Targeted deep sequencing was performed after protoplasts were incubated for 7 days. Converted nucleotides in the target spacer region are shown in red.

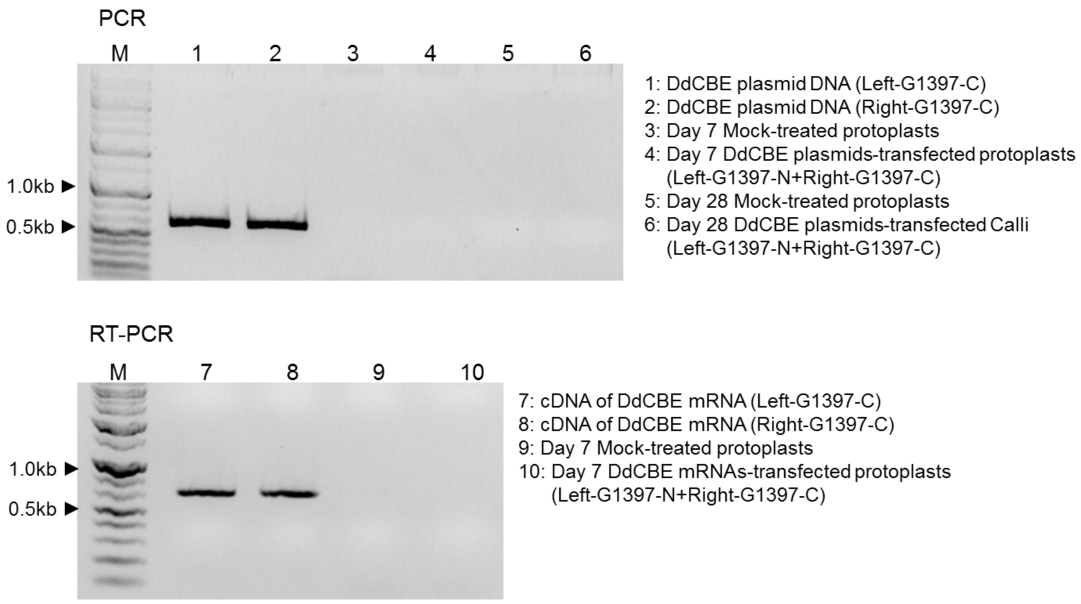

Supplementary Figure 7. Absence of DdCBE mRNA or DNA sequences in protoplasts and calli. PCR and RT-PCR were used to confirm the absence of DdCBE-encoding DNA and mRNA sequences, respectively, in protoplasts and calli at day 7 and 28 post-transfection. We obtained similar results more than three times when the gel electrophoresis was repeated independently. “M” represents size marker.

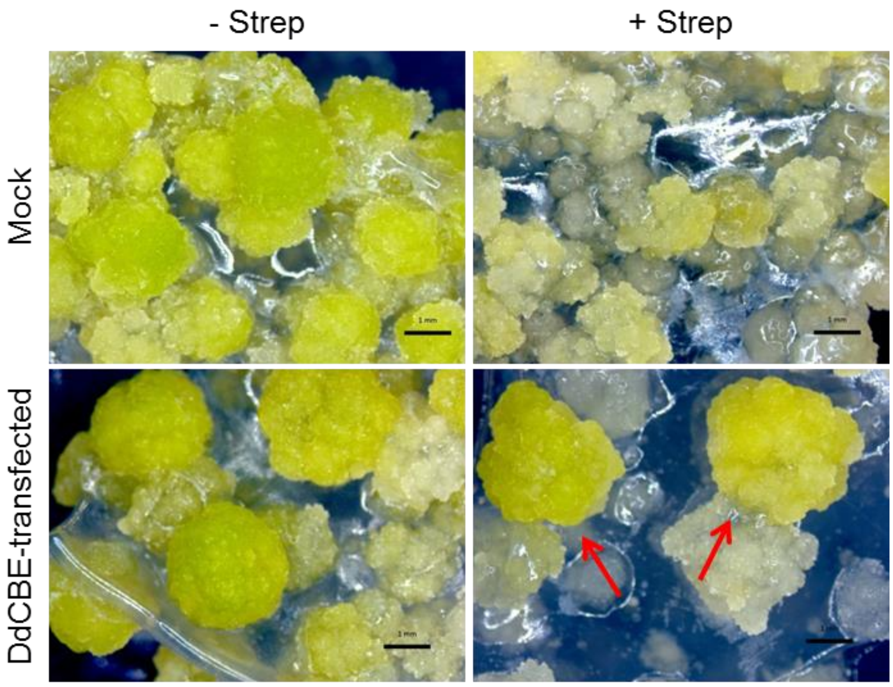

Supplementary Figure 8. Selection of 16s rDNA mutants. The red arrows indicate streptomycin-resistant greenish calli.

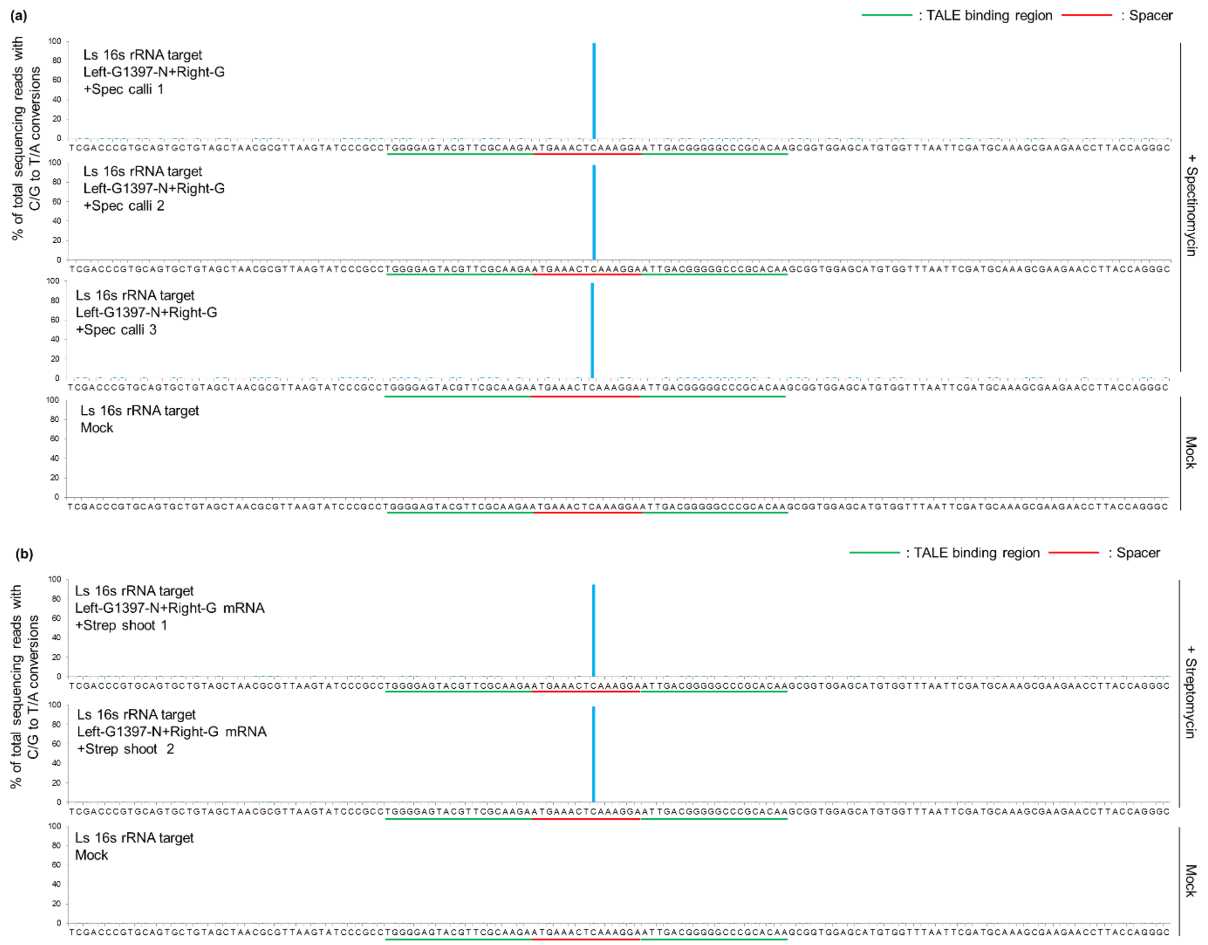

Supplementary Figure 9. No off-target mutations were detected in the immediate proximity of the DdCBE target site in antibiotic-resistant calli or plantlets. (a), (b) Off-target activity was analyzed by targeted deep sequencing. TALE binding sites and the spacer region are indicated by green and red underbars, respectively. (a) Spectinomycin-resistant calli cultured from DdCBE plasmid-transfected lettuce protoplasts. (b) Shoots obtained from a streptomycin-resistant plantlet.

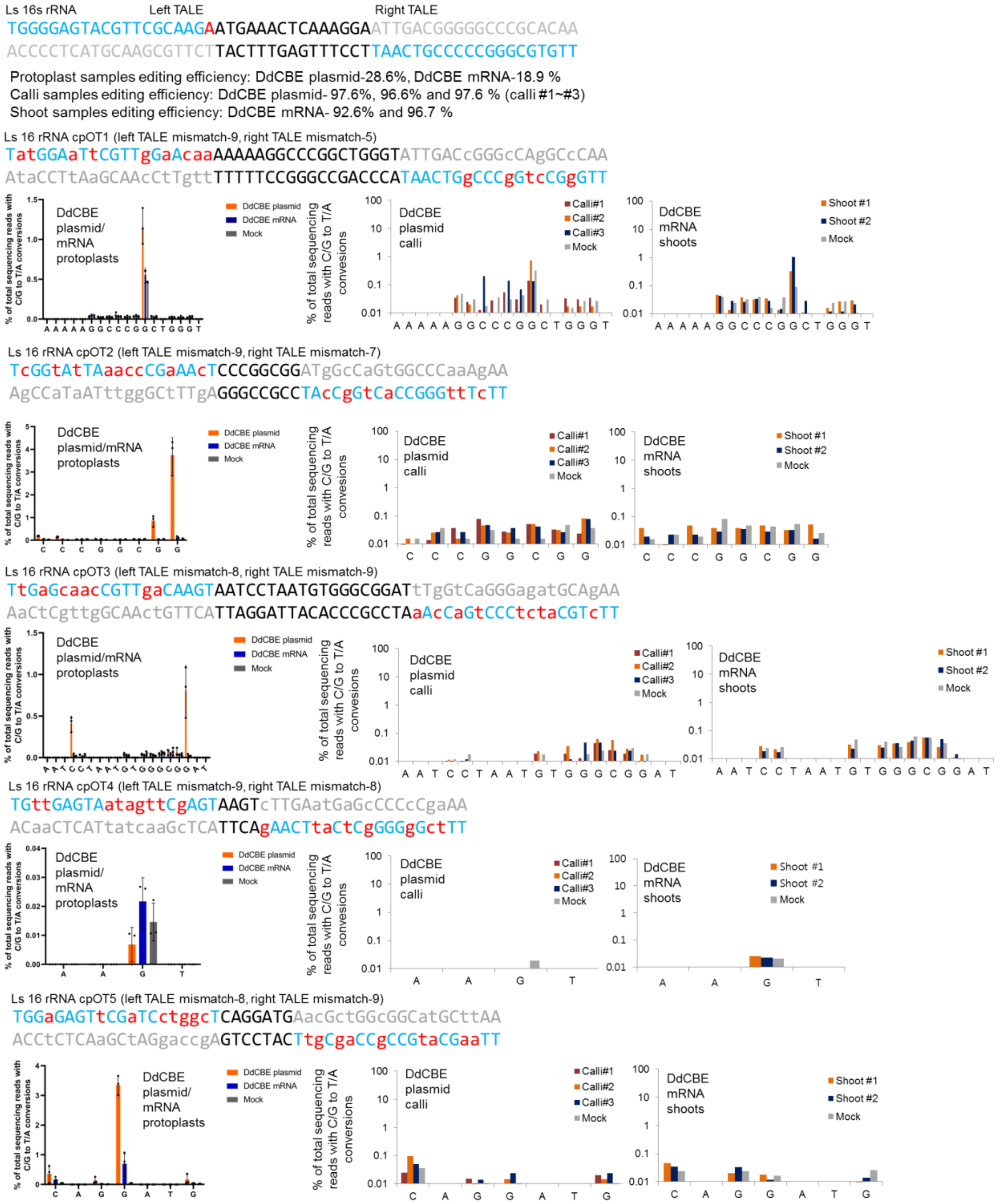

Supplementary Figure 10. Analysis of off-target activity at the 5 sites with the most homology with the on-target site. Five potential off-target sites of the 16s rRNA gene-specific DdCBE in the lettuce chloroplast genome, with up to 9 mismatches in the TALE-binding site, were chosen for this analysis. TALE binding

sequences and mismatched nucleotides are shown in blue and red, respectively. Off-target mutation frequencies were measured in DdCBE plasmid- or DdCBE mRNA-transfected protoplasts and drug-resistant calli or shoots using targeted deep sequencing. Frequencies (mean±s.d.) were obtained from three independent experiments.

Supplementary Table 1. PCR primers used in this study.

Primer name	Primer sequence (5'-3')	Purpose
16s nested F	GCTCGCGTCTGATTAGCTAG	Targeted deep sequencing 16s rDNA 1st PCR
16s nested R	AACGAATTCACCGCCGTATG	Targeted deep sequencing 16s rDNA 1st PCR
16s 2nd F	ACACTCTTTCCCTACACGACGCTCTTCCGATCT CGTATCGACCCGTGCAGT	Targeted deep sequencing 16s rDNA 2nd PCR
16s 2nd R	GACTGGAGTTCAGACGTGTGCTCTTCCGATCT GGCACCCCTCTCTTTCAAGA	Targeted deep sequencing 16s rDNA 2nd PCR
psbA nested F	GACTGCAATTTTAGAGAGACGC	Targeted deep sequencing *pbsA* 1st PCR
psbA nested R	CGTTCATGCATAACTTCCATACC	Targeted deep sequencing *pbsA* 1st PCR
psbA 2nd Bn F	ACACTCTTTCCCTACACGACGCTCTTCCGATCT ATTGTTCTACACTTTTTACTTGG	Targeted deep sequencing *pbsA* 2nd PCR in rapeseed
psbA 2nd Bn, Ls R	GACTGGAGTTCAGACGTGTGCTCTTCCGATCT ATTCCTAGAGGCATACCATC	Targeted deep sequencing *pbsA* 2nd PCR
pbsA 2nd Ls F	ACACTCTTTCCCTACACGACGCTCTTCCGATCT TTGTTCTACACTTCTTACTTGG	Targeted deep sequencing *pbsA* 2nd PCR in lettuce
psbB nested F	GGTTTGCCTTGGTATCGTGT	Targeted deep sequencing *psbB* 1st PCR
psbB nested R	ACTCAACAGTTACACCTACTTGT	Targeted deep sequencing *psbB* 1st PCR
psbB 2nd F	ACACTCTTTCCCTACACGACGCTCTTCCGATCT TTGGCATTGGGTATATTGGGA	Targeted deep sequencing *psbB* 2nd PCR
psbB 2nd R	GACTGGAGTTCAGACGTGTGCTCTTCCGATCT TCGGATACCCATATTCCAGGAC	Targeted deep sequencing *psbB* 2nd

		PCR
atp6 Bn nested F	GTCCCCACTTGACCAATTTGA	Targeted deep sequencing *atp6* 1st PCR in rapeseed
atp6 Bn nested R	ATAGTCCAAGCGAACCCACT	Targeted deep sequencing *atp6* 1st PCR in rapeseed
atp6 Bn 2nd F	ACACTCTTTCCCTACACGACGCTCTTCCGATCT TAGCTTCACAGTGACAAGTC	Targeted deep sequencing *atp6* 2nd PCR in rapeseed
atp6 Bn 2nd R	GACTGGAGTTCAGACGTGTGCTCTTCCGATCT GCTTAATGCGCGAAAACAAT	Targeted deep sequencing *atp6* 2nd PCR in rapeseed
atp6 Ls nested F	CGATTACGCCCAACAGCC	Targeted deep sequencing *atp6* 1st PCR in lettuce
atp6 Ls nested R	GCTACACCTAATTCCAGACCG	Targeted deep sequencing *atp6* 1st PCR in lettuce
atp6 Ls 2nd F	ACACTCTTTCCCTACACGACGCTCTTCCGATCT TAGCTTCACAGTTACAAGTC	Targeted deep sequencing *atp6* 2nd PCR in lettuce
atp6 Ls 2nd R	GACTGGAGTTCAGACGTGTGCTCTTCCGATCT GCTTAATGCGCGAAAACAAT	Targeted deep sequencing *atp6* 2nd PCR in lettuce
rps14 nested F	ACACAAGATGAGACTTTACCACT	Targeted deep sequencing *rps14* 1st PCR
rps14 nested R	GGGAGCATACTTGACAGGAAA	Targeted deep sequencing *rps14* 1st PCR
rps14 2nd F	ACACTCTTTCCCTACACGACGCTCTTCCGATCT AGTTGTCCAAGTTGCCAAGA	Targeted deep sequencing *rps14* 2nd PCR
rps14 2nd R	GACTGGAGTTCAGACGTGTGCTCTTCCGATCT TCTATTGGTTTGGTGGTTGCT	Targeted deep sequencing *rps14* 2nd PCR
mRNA F	TTCATCTCATCCGTTTAGAAGCT	mRNA IVT template
mRNA R	CCATTTGCATTTTGATGTCCGA	mRNA IVT template

16s cpOT1 nested F	GGGGTTTGCGAAATTTGAAAGA	Targeted deep sequencing 16s cpOT1 1st PCR
16s cpOT1 nested R	AGTTTATTGTACTTGCATCCCTTTT	Targeted deep sequencing 16s cpOT1 1st PCR
16s cpOT1 2nd F	ACACTCTTTCCCTACACGACGCTCTTCCGATCT ACCTTCTTGCTTTTATTTTGTTCGA	Targeted deep sequencing 16s cpOT1 2nd PCR
16s cpOT1 2nd R	GACTGGAGTTCAGACGTGTGCTCTTCCGATCT ACGATAAGTATATGTAAACCCCACA	Targeted deep sequencing 16s cpOT1 2nd PCR
16s cpOT2 nested F	CCTTGGACTTGTGCTTTGCT	Targeted deep sequencing 16s cpOT2 1st PCR
16s cpOT2 nested R	ACCTAGGTCATCGATTCAGCA	Targeted deep sequencing 16s cpOT2 1st PCR
16s cpOT2 2nd F	ACACTCTTTCCCTACACGACGCTCTTCCGATCT GAGGGCAAAGTAATACAAAAAGAAC	Targeted deep sequencing 16s cpOT2 2nd PCR
16s cpOT2 2nd R	GACTGGAGTTCAGACGTGTGCTCTTCCGATCT GTTGGATCCAGCCTATTCTTGA	Targeted deep sequencing 16s cpOT2 2nd PCR
16s cpOT3 nested F	TCCTATTCTTTCCCCGGACC	Targeted deep sequencing 16s cpOT3 1st PCR
16s cpOT3 nested R	ACTGTGGCTAGGAATGTGGT	Targeted deep sequencing 16s cpOT3 1st PCR
16s cpOT3 2nd F	ACACTCTTTCCCTACACGACGCTCTTCCGATCT CGGATGGACGTCAAGTGGTT	Targeted deep sequencing 16s cpOT3 2nd PCR
16s cpOT3 2nd R	GACTGGAGTTCAGACGTGTGCTCTTCCGATCT TTGTGCCAAAATAACAGATGCT	Targeted deep sequencing 16s cpOT3 2nd PCR
16s cpOT4 nested F	GTTCATTCTCCGGGCAACTC	Targeted deep sequencing 16s cpOT4 1st PCR
16s cpOT4 nested R	TGAAGTAAAGGCTCCGGTGT	Targeted deep sequencing 16s cpOT4 1st PCR

16s cpOT4 2nd F	ACACTCTTTCCCTACACGACGCTCTTCCGATCT GCTGCATTTATTCGAGTGATCC	Targeted deep sequencing 16s cpOT4 2nd PCR
16s cpOT4 2nd R	GACTGGAGTTCAGACGTGTGCTCTTCCGATCT TGACCAGAATTAGACGGGGA	Targeted deep sequencing 16s cpOT4 2nd PCR
16s cpOT5 nested F	TGTGGCTGATCATCCTCTCG	Targeted deep sequencing 16s cpOT5 1st PCR
16s cpOT5 nested R	TCTCGACCCTTTGCCTTAGG	Targeted deep sequencing 16s cpOT5 1st PCR
16s cpOT5 2nd F	ACACTCTTTCCCTACACGACGCTCTTCCGATCT CGTTTCCAGCTGTTGTTCCC	Targeted deep sequencing 16s cpOT5 2nd PCR
16s cpOT5 2nd R	GACTGGAGTTCAGACGTGTGCTCTTCCGATCT CCGAATCCGCTTTGTCTACG	Targeted deep sequencing 16s cpOT5 2nd PCR
DdCBE PCR and RT-PCR F	CCTCTATGCTCTCTTCCGCTA	PCR and RT-PCR
DdCBE PCR and RT-PCR R	AGAGTTGTCCAGTATCAAGCTG	PCR and RT-PCR

Supplementary sequences 1. DNA sequences of plasmid components.

Parsley ubiquitin promoter
CTAGCAACGATTGTACAATTGCTTCTTTAAAAAAGGAAGAAAGAAAGAAAGAAAAGAATC
AACATCAGCGTTAACAAACGGCCCCGTTACGGCCCAAACGGTCATATAGAGTAACGGC
GTTAAGCGTTGAAAGACTCCTATCGAAATACGTAACCGCAAACGTGTCATAGTCAGATC
CCCTCTTCCTTCACCGCCTCAAACACAAAAATAATCTTCTACAGCCTATATATACAACCC
CCCCTTCTATCTCTCCTTTCTCACAATTCATCATCTTTCTTTCTCTACCCCCAATTTTAAG
AAATCCTCTCTTCTCCTCTTCATTTTCAAGGTAAATCTCTCTCTCTCTCTCTCTCTCTGTT
ATTCCTTGTTTTAATTAGGTATGTATTATTGCTAGTTTGTTAATCTGCTTATCTTATGTATG
CCTTATGTGAATATCTTTATCTTGTTCATCTCATCCGTTTAGAAGCTATAAATTTGTTGAT
TTGACTGTGTATCTACACGTGGTTATGTTTATATCTAATCAGATATGAATTTCTTCATATT
GTTGCGTTTGTGTGTACCAATCCGAAATCGTTGATTTTTTTCATTTAATCGTGTAGCTAAT
TGTACGTATACATATGGATCTACGTATCAATTGTTCATCTGTTTGTGTTTGTATGTATACA
GATCTGAAAACATCACTTCTCTCATCTGATTGTGTTGTTACATACATAGATATAGATCTGT
TATATCATTTTTTTTATTAATTGTGTATATATATATGTGCATAGATCTGGATTACATGATTG
TGATTATTTACATGATTTTGTTATTTACGTATGTATATATGTAGATCTGGACTTTTTGGAG
TTGTTGACTTGATTGTATTTGTGTGTGTATATGTGTGTTCTGATCTTGATATGTTATGTAT
GTGCAGC

T7 promoter
TAATACGACTCACTATAGG

pea3A terminator
CAGGCCTCCCAGCTTTCGTCCGTATCATCGGTTTCGACAACGTTCGTCAAGTTCAATGC
ATCAGTTTCATTGCCCACACACCAGAATCCTACTAAGTTTGAGTATTATGGCATTGGAAA
AGCTGTTTTCTTCTATCATTTGTTCTGCTTGTAATTTACTGTGTTCTTTCAGTTTTTGTTTT
CGGACATCAAAATGCAAATGGATGGATAAGAGTTAATAAATGATATGGTCCTTTTGTTCA
TTCTCAAATTATTATTATCTGTTGTTTTTACTTTAATGGGTTGAATTTAAGTAAGAAAGGA
ACTAACAGTGTGATATTAAGGTGCAATGTTAGACATATAAAACAGTCTTTCACCTCTCTT
TGGTTATGTCTTGAATTGGTTTGTTTCTTCACTTATCTGTGTAATCAAGTTTACTATGAGT
CTATGATCAAGTAATTATGCAATCAAGTTAAGTACAGTATAGGCTT

Supplementary sequences 2. Amino acid sequences of DdCBE components.

AtinfA CTP-3xHA-N terminal domain
MLQLCSTFRPQLLLPCQFRFTNGVLIPQINYVASNSVVNIRPMIRCQRASGGRGGANRSKPAK
PQVKEGSNKTVIEGLVTESLPNGMFRVDLENGDNILGYICGKIRKNFIRILPGDKVKVEMSVYDS
TKGRIIFRMSSRDYPYDVPDYAGYPYDVPDYAGYPYDVPDYAMDIADLRTLGYSQQQQEKIKP
KVRSTVAQHHEALVGHGFTHAHIVALSQHPAALGTVAVKYQDMIAALPEATHEAIVGVGKQWS
GARALEALLTVAGELRGPPLQLDTGQLLKIAKRGGVTAVEAVHAWRNALTGAPL

AtRbcS CTP-3xFLAG-N terminal domain
MASSMLSSATMVASPAQATMVAPFNGLKSSAAFPATRKANNDITSITSNGGRVNCMQVWPPI
GKKKFETLSYLPDLTDSEDYKDHDGDYKDHDIDYKDDDDKMDIADLRTLGYSQQQQEKIKPKV
RSTVAQHHEALVGHGFTHAHIVALSQHPAALGTVAVKYQDMIAALPEATHEAIVGVGKRGAGA
RALEALLTVAGELRGPPLQLDTGQLLKIAKRGGVTAVEAVHAWRNALTGAPL

AtATPase gamma subunit MTS-3xHA-N terminal domain
MAMAVFRREGRRLLPSIAARPIAAIRSPLSSDQEEGLLGVRSISTQVVRNRMKSVKNIQKITKAM
KMVAASKLRAVQYPYDVPDYAGYPYDVPDYAGYPYDVPDYAMDIADLRTLGYSQQQQEKIKP
KVRSTVAQHHEALVGHGFTHAHIVALSQHPAALGTVAVKYQDMIAALPEATHEAIVGVGKQWS
GARALEALLTVAGELRGPPLQLDTGQLLKIAKRGGVTAVEAVHAWRNALTGAPL

AtATPase delta subunit MTS-3xFLAG-N terminal domain
MFKQASRLLSRSVAAASSKSVTTRAFSTELPSTLDSDYKDHDGDYKDHDIDYKDDDDKMDIAD
LRTLGYSQQQQEKIKPKVRSTVAQHHEALVGHGFTHAHIVALSQHPAALGTVAVKYQDMIAAL
PEATHEAIVGVGKRGAGARALEALLTVAGELRGPPLQLDTGQLLKIAKRGGVTAVEAVHAWRN
ALTGAPL

Half (**NG**) domain-C-terminal domain-G1333-N-UGI
GLTPEQVVAIAS**NG**GGKQALESIVAQLSRPDPALAALTNDHLVALACLGGRPALDAVKKGLGG
SGSYALGPYQISAPQLPAYNGQTVGTFYYVNDAGGLESKVFSSGGSGGSTNLSDIIEKETGKQ
LVIQESILMLPEEVEEVIGNKPESDILVHTAYDESTDENVMLLTSDAPEYKPWALVIQDSNGENKI
KML*

Half (**NG**) domain-C-terminal domain-G1333-C-UGI
GLTPEQVVAIAS**NG**GGKQALESIVAQLSRPDPALAALTNDHLVALACLGGRPALDAVKKGLGG
SPTPYPNYANAGHVEGQSALFMRDNGISEGLVFHNNPEGTCGFCVNMTETLLPENAKMTVVP
PEGAIPVKRGATGETKVFTGNSNSPKSPTKGGCSGGSTNLSDIIEKETGKQLVIQESILMLPEEV
EEVIGNKPESDILVHTAYDESTDENVMLLTSDAPEYKPWALVIQDSNGENKIKML*

Half (**NG**) domain-C-terminal domain-G1397-N-UGI
GLTPEQVVAIAS**NG**GGKQALESIVAQLSRPDPALAALTNDHLVALACLGGRPALDAVKKGLGG
SGSYALGPYQISAPQLPAYNGQTVGTFYYVNDAGGLESKVFSSGGPTPYPNYANAGHVEGQS
ALFMRDNGISEGLVFHNNPEGTCGFCVNMTETLLPENAKMTVVPPEGSGGSTNLSDIIEKETG
KQLVIQESILMLPEEVEEVIGNKPESDILVHTAYDESTDENVMLLTSDAPEYKPWALVIQDSNGE
NKIKML*

Half (**NG**) domain-C-terminal domain-G1397-C-UGI
GLTPEQVVAIAS**NG**GGKQALESIVAQLSRPDPALAALTNDHLVALACLGGRPALDAVKKGLGG
SAIPVKRGATGETKVFTGNSNSPKSPTKGGCSGGSTNLSDIIEKETGKQLVIQESILMLPEEVEE
VIGNKPESDILVHTAYDESTDENVMLLTSDAPEYKPWALVIQDSNGENKIKML*

Supplementary sequences 3. Amino acid sequences of TALE repeats.

16s rDNA Left TALE repeat - GGGGAGTACGTTCGCAAG
NLTPAQVVAIASNNGGKQALETVQRLLPVLCQAHGLTPDQVVAIASNNGGKQALETVQRLLPVLCQDH
GLTPEQVVAIASNNGGKQALETVQRLLPVLCQAHGLTPDQVVAIASNNGGKQALETVQRLLPVLCQDH
GLTPAQVVAIASNIGGKQALETVQRLLPVLCQAHGLTPEQVVAIASNNGGKQALETVQRLLPVLCQAH
GLTPEQVVAIASNGGGKQALETVQRLLPVLCQAHGLTPDQVVAIASNIGGKQALETVQRLLPVLCQAH
GLTPAQVVAIASHDGGKQALETVQRLLPVLCQAHGLTPAQVVAIASNNGGKQALETVQRLLPVLCQDH
GLTPEQVVAIASNGGGKQALETVQRLLPVLCQAHGLTPAQVVAIASNGGGKQALETVQRLLPVLCQDH
GLTPAQVVAIASHDGGKQALETVQRLLPVLCQAHGLTPDQVVAIASNNGGKQALETVQRLLPVLCQDH
GLTPDQVVAIASHDGGKQALETVQRLLPVLCQDHGLTPAQVVAIASNIGGKQALETVQRLLPVLCQDH
GLTPDQVVAIASNIGGKQALETVQRLLPVLCQDHGLTPDQVVAIASNNGGKQALETVQRLLPVLCQAH

16s rDNA Right TALE repeat - TGTGCGGGCCCCCGTCAA
NLTPAQVVAIASNGGGKQALETVQRLLPVLCQAHGLTPDQVVAIASNNGGKQALETVQRLLPVLCQAH
GLTPAQVVAIASNGGGKQALETVQRLLPVLCQDHGLTPAQVVAIASNNGGKQALETVQRLLPVLCQAH
GLTPDQVVAIASHDGGKQALETVQRLLPVLCQDHGLTPAQVVAIASNNGGKQALETVQRLLPVLCQAH
GLTPEQVVAIASNNGGKQALETVQRLLPVLCQAHGLTPAQVVAIASNNGGKQALETVQRLLPVLCQDH
GLTPDQVVAIASHDGGKQALETVQRLLPVLCQAHGLTPAQVVAIASHDGGKQALETVQRLLPVLCQAH
GLTPDQVVAIASHDGGKQALETVQRLLPVLCQDHGLTPDQVVAIASHDGGKQALETVQRLLPVLCQDH
GLTPDQVVAIASHDGGKQALETVQRLLPVLCQDHGLTPDQVVAIASNNGGKQALETVQRLLPVLCQDH
GLTPAQVVAIASNGGGKQALETVQRLLPVLCQAHGLTPAQVVAIASHDGGKQALETVQRLLPVLCQDH
GLTPEQVVAIASNIGGKQALETVQRLLPVLCQAHGLTPAQVVAIASNIGGKQALETVQRLLPVLCQDH

psbA Left TALE repeat - GCAACAGGAGCTGAATAT
NLTPAQVVAIASNNGGKQALETVQRLLPVLCQAHGLTPDQVVAIASHDGGKQALETVQRLLPVLCQDH
GLTPAQVVAIASNIGGKQALETVQRLLPVLCQAHGLTPDQVVAIASNIGGKQALETVQRLLPVLCQDH
GLTPDQVVAIASHDGGKQALETVQRLLPVLCQDHGLTPAQVVAIASNIGGKQALETVQRLLPVLCQAH
GLTPAQVVAIASNNGGKQALETVQRLLPVLCQAHGLTPDQVVAIASNNGGKQALETVQRLLPVLCQDH
GLTPAQVVAIASNIGGKQALETVQRLLPVLCQAHGLTPDQVVAIASNNGGKQALETVQRLLPVLCQAH
GLTPAQVVAIASHDGGKQALETVQRLLPVLCQDHGLTPDQVVAIASNGGGKQALETVQRLLPVLCQAH
GLTPEQVVAIASNNGGKQALETVQRLLPVLCQAHGLTPDQVVAIASNIGGKQALETVQRLLPVLCQDH
GLTPDQVVAIASNIGGKQALETVQRLLPVLCQAHGLTPDQVVAIASNGGGKQALETVQRLLPVLCQAH
GLTPDQVVAIASNIGGKQALETVQRLLPVLCQDHGLTPDQVVAIASNGGGKQALETVQRLLPVLCQAH

psbA Right TALE repeat - AGTTTCCGTCTGGGTATG
NLTPAQVVAIASNIGGKQALETVQRLLPVLCQAHGLTPEQVVAIASNNGGKQALETVQRLLPVLCQAH
GLTPDQVVAIASNGGGKQALETVQRLLPVLCQDHGLTPDQVVAIASNGGGKQALETVQRLLPVLCQDH
GLTPEQVVAIASNGGGKQALETVQRLLPVLCQAHGLTPDQVVAIASHDGGKQALETVQRLLPVLCQAH
GLTPDQVVAIASHDGGKQALETVQRLLPVLCQDHGLTPDQVVAIASNNGGKQALETVQRLLPVLCQDH
GLTPAQVVAIASNGGGKQALETVQRLLPVLCQAHGLTPAQVVAIASHDGGKQALETVQRLLPVLCQAH
GLTPAQVVAIASNGGGKQALETVQRLLPVLCQDHGLTPDQVVAIASNNGGKQALETVQRLLPVLCQDH
GLTPAQVVAIASNNGGKQALETVQRLLPVLCQDHGLTPAQVVAIASNNGGKQALETVQRLLPVLCQDH
GLTPDQVVAIASNGGGKQALETVQRLLPVLCQAHGLTPEQVVAIASNIGGKQALETVQRLLPVLCQAH
GLTPAQVVAIASNGGGKQALETVQRLLPVLCQAHGLTPAQVVAIASNNGGKQALETVQRLLPVLCQDH

psbB Left TALE repeat - AAATGAATTCCAAAAATC
NLTPDQVVAIASNIGGKQALETVQRLLPVLCQAHGLTPAQVVAIASNIGGKQALETVQRLLPVLCQDH
GLTPAQVVAIASNIGGKQALETVQRLLPVLCQAHGLTPAQVVAIASNGGGKQALETVQRLLPVLCQAH
GLTPAQVVAIASNNGGKQALETVQRLLPVLCQDHGLTPAQVVAIASNIGGKQALETVQRLLPVLCQDH
GLTPAQVVAIASNIGGKQALETVQRLLPVLCQAHGLTPDQVVAIASNGGGKQALETVQRLLPVLCQAH
GLTPDQVVAIASNGGGKQALETVQRLLPVLCQAHGLTPAQVVAIASHDGGKQALETVQRLLPVLCQDH
GLTPDQVVAIASHDGGKQALETVQRLLPVLCQAHGLTPDQVVAIASNIGGKQALETVQRLLPVLCQAH
GLTPDQVVAIASNIGGKQALETVQRLLPVLCQAHGLTPAQVVAIASNIGGKQALETVQRLLPVLCQDH
GLTPAQVVAIASNIGGKQALETVQRLLPVLCQAHGLTPDQVVAIASNIGGKQALETVQRLLPVLCQDH
GLTPAQVVAIASNGGGKQALETVQRLLPVLCQAHGLTPAQVVAIASHDGGKQALETVQRLLPVLCQDH

psbB Right TALE repeat - GAACGTACAGGAAAACC
NLTPEQVVAIASNNGGKQALETVQRLLPVLCQAHGLTPDQVVAIASNIGGKQALETVQRLLPVLCQDH
GLTPDQVVAIASNIGGKQALETVQRLLPVLCQAHGLTPDQVVAIASHDGGKQALETVQRLLPVLCQDH
GLTPDQVVAIASNNGGKQALETVQRLLPVLCQDHGLTPAQVVAIASNGGGKQALETVQRLLPVLCQAH
GLTPDQVVAIASNIGGKQALETVQRLLPVLCQDHGLTPDQVVAIASHDGGKQALETVQRLLPVLCQDH
GLTPAQVVAIASNIGGKQALETVQRLLPVLCQAHGLTPAQVVAIASNNGGKQALETVQRLLPVLCQAH
GLTPDQVVAIASNNGGKQALETVQRLLPVLCQDHGLTPAQVVAIASNIGGKQALETVQRLLPVLCQAH
GLTPDQVVAIASNIGGKQALETVQRLLPVLCQAHGLTPAQVVAIASNIGGKQALETVQRLLPVLCQDH
GLTPAQVVAIASNIGGKQALETVQRLLPVLCQAHGLTPAQVVAIASHDGGKQALETVQRLLPVLCQDH
GLTPAQVVAIASHDGGKQALETVQRLLPVLCQAH

ATP6 (lettuce, rapeseed) Left TALE repeat - TATTGGCATTACTATAG
NLTPDQVVAIASNGGGKQALETVQRLLPVLCQAHGLTPDQVVAIASNIGGKQALETVQRLLPVLCQDH
GLTPDQVVAIASNGGGKQALETVQRLLPVLCQAHGLTPEQVVAIASNGGGKQALETVQRLLPVLCQAH
GLTPAQVVAIASNNGGKQALETVQRLLPVLCQDHGLTPDQVVAIASNNGGKQALETVQRLLPVLCQDH
GLTPAQVVAIASHDGGKQALETVQRLLPVLCQDHGLTPDQVVAIASNIGGKQALETVQRLLPVLCQDH
GLTPAQVVAIASNGGGKQALETVQRLLPVLCQAHGLTPEQVVAIASNGGGKQALETVQRLLPVLCQAH
GLTPDQVVAIASNIGGKQALETVQRLLPVLCQAHGLTPAQVVAIASHDGGKQALETVQRLLPVLCQDH
GLTPDQVVAIASNGGGKQALETVQRLLPVLCQAHGLTPDQVVAIASNIGGKQALETVQRLLPVLCQDH
GLTPDQVVAIASNGGGKQALETVQRLLPVLCQAHGLTPDQVVAIASNIGGKQALETVQRLLPVLCQDH
GLTPAQVVAIASNNGGKQALETVQRLLPVLCQDH

ATP6 (lettuce) Right TALE repeat - GAAAAAATGAAGCCCA
NLTPEQVVAIASNGGGKQALETVQRLLPVLCQAHGLTPAQVVAIASNIGGKQALETVQRLLPVLCQDH
GLTPAQVVAIASNIGGKQALETVQRLLPVLCQAHGLTPDQVVAIASNIGGKQALETVQRLLPVLCQAH
GLTPAQVVAIASNIGGKQALETVQRLLPVLCQDHGLTPAQVVAIASNIGGKQALETVQRLLPVLCQAH
GLTPEQVVAIASNIGGKQALETVQRLLPVLCQAHGLTPAQVVAIASNGGGKQALETVQRLLPVLCQAH
GLTPAQVVAIASNNGGKQALETVQRLLPVLCQAHGLTPAQVVAIASNIGGKQALETVQRLLPVLCQDH
GLTPDQVVAIASNIGGKQALETVQRLLPVLCQDHGLTPDQVVAIASNNGGKQALETVQRLLPVLCQAH
GLTPAQVVAIASHDGGKQALETVQRLLPVLCQAHGLTPDQVVAIASHDGGKQALETVQRLLPVLCQDH
GLTPDQVVAIASHDGGKQALETVQRLLPVLCQDHGLTPDQVVAIASNIGGKQALETVQRLLPVLCQAH

ATP6 (rapeseed) Right TALE repeat - TAAAAAATGAAGCCCA
NLTPEQVVAIASNNGGKQALETVQRLLPVLCQAHGLTPDQVVAIASNIGGKQALETVQRLLPVLCQDH
GLTPDQVVAIASNIGGKQALETVQRLLPVLCQAHGLTPDQVVAIASNIGGKQALETVQRLLPVLCQAH
GLTPAQVVAIASNIGGKQALETVQRLLPVLCQDHGLTPAQVVAIASNIGGKQALETVQRLLPVLCQAH
GLTPEQVVAIASNIGGKQALETVQRLLPVLCQAHGLTPAQVVAIASNGGGKQALETVQRLLPVLCQAH
GLTPAQVVAIASNNGGKQALETVQRLLPVLCQAHGLTPAQVVAIASNIGGKQALETVQRLLPVLCQDH
GLTPDQVVAIASNIGGKQALETVQRLLPVLCQDHGLTPDQVVAIASNNGGKQALETVQRLLPVLCQAH
GLTPAQVVAIASHDGGKQALETVQRLLPVLCQDHGLTPAQVVAIASHDGGKQALETVQRLLPVLCQAH
GLTPAQVVAIASHDGGKQALETVQRLLPVLCQDHGLTPDQVVAIASNIGGKQALETVQRLLPVLCQAH

RPS14 Left TALE repeat - CGTTCTGTATCTGAG
NLTPDQVVAIASHDGGKQALETVQRLLPVLCQDHGLTPDQVVAIASNNGGKQALETVQRLLPVLCQDH
GLTPAQVVAIASNGGGKQALETVQRLLPVLCQAHGLTPEQVVAIASNGGGKQALETVQRLLPVLCQAH
GLTPDQVVAIASHDGGKQALETVQRLLPVLCQDHGLTPDQVVAIASNGGGKQALETVQRLLPVLCQAH
GLTPDQVVAIASNNGGKQALETVQRLLPVLCQAHGLTPAQVVAIASNGGGKQALETVQRLLPVLCQDH
GLTPAQVVAIASNIGGKQALETVQRLLPVLCQAHGLTPEQVVAIASNGGGKQALETVQRLLPVLCQAH
GLTPDQVVAIASHDGGKQALETVQRLLPVLCQDHGLTPDQVVAIASNGGGKQALETVQRLLPVLCQAH
GLTPAQVVAIASNNGGKQALETVQRLLPVLCQAHGLTPAQVVAIASNIGGKQALETVQRLLPVLCQAH
GLTPDQVVAIASNNGGKQALETVQRLLPVLCQAH

RPS14 Right TALE repeat - AATCCACGAAAAACGA
NLTPDQVVAIASNIGGKQALETVQRLLPVLCQAHGLTPDQVVAIASNIGGKQALETVQRLLPVLCQDH
GLTPDQVVAIASNGGGKQALETVQRLLPVLCQAHGLTPAQVVAIASHDGGKQALETVQRLLPVLCQDH
GLTPDQVVAIASHDGGKQALETVQRLLPVLCQAHGLTPDQVVAIASNIGGKQALETVQRLLPVLCQAH
GLTPEQVVAIASHDGGKQALETVQRLLPVLCQAHGLTPAQVVAIASNNGGKQALETVQRLLPVLCQAH
GLTPAQVVAIASNIGGKQALETVQRLLPVLCQAHGLTPDQVVAIASNIGGKQALETVQRLLPVLCQAH
GLTPAQVVAIASNIGGKQALETVQRLLPVLCQDHGLTPAQVVAIASNIGGKQALETVQRLLPVLCQAH
GLTPDQVVAIASNIGGKQALETVQRLLPVLCQDHGLTPDQVVAIASHDGGKQALETVQRLLPVLCQAH
GLTPAQVVAIASNNGGKQALETVQRLLPVLCQDHGLTPDQVVAIASNIGGKQALETVQRLLPVLCQAH
