# Supplementary material for: Chloroplast and mitochondrial DNA editing in plants
Source: Nat Plants. 2021 Jul 1;7(7):899–905. doi: 10.1038/s41477-021-00943-9 (PMC8289734; doi:10.1038/s41477-021-00943-9)
Supplement: Supplementary file 2 — Reporting Summary [file 41477_2021_943_MOESM2_ESM.pdf]

nature research

Corresponding author(s): Jin-Soo Kim

Last updated by author(s): May 8, 2021

Reporting Summary

Nature Research wishes to improve the reproducibility of the work that we publish. This form provides structure for consistency and transparency in reporting. For further information on Nature Research policies, see our Editorial Policies and the Editorial Policy Checklist.

Statistics

For all statistical analyses, confirm that the following items are present in the figure legend, table legend, main text, or Methods section.

n/a	Confirmed	
☐	☒	The exact sample size (n) for each experimental group/condition, given as a discrete number and unit of measurement
☐	☒	A statement on whether measurements were taken from distinct samples or whether the same sample was measured repeatedly
☐	☒	The statistical test(s) used AND whether they are one- or two-sided<br>*Only common tests should be described solely by name; describe more complex techniques in the Methods section.*
☒	☐	A description of all covariates tested
☒	☐	A description of any assumptions or corrections, such as tests of normality and adjustment for multiple comparisons
☐	☒	A full description of the statistical parameters including central tendency (e.g. means) or other basic estimates (e.g. regression coefficient) AND variation (e.g. standard deviation) or associated estimates of uncertainty (e.g. confidence intervals)
☐	☒	For null hypothesis testing, the test statistic (e.g. F, t, r) with confidence intervals, effect sizes, degrees of freedom and P value noted<br>*Give P values as exact values whenever suitable.*
☒	☐	For Bayesian analysis, information on the choice of priors and Markov chain Monte Carlo settings
☒	☐	For hierarchical and complex designs, identification of the appropriate level for tests and full reporting of outcomes
☒	☐	Estimates of effect sizes (e.g. Cohen's d, Pearson's r), indicating how they were calculated

Our web collection on statistics for biologists contains articles on many of the points above.

Software and code

Policy information about availability of computer code

Data collection	Illumina Miniseq platform was used to collect the targeted deep sequencing data.
Data analysis	High-throughput sequencing data was analyzed using Cas-analyzer (reference 21) and source code of the computer program at https://github.com/ibs-cge/maund. Graphpad prism 9 was used to analyze the data.

For manuscripts utilizing custom algorithms or software that are central to the research but not yet described in published literature, software must be made available to editors and reviewers. We strongly encourage code deposition in a community repository (e.g. GitHub). See the Nature Research guidelines for submitting code & software for further information.

Data

Policy information about availability of data

All manuscripts must include a data availability statement. This statement should provide the following information, where applicable:

- Accession codes, unique identifiers, or web links for publicly available datasets
- A list of figures that have associated raw data
- A description of any restrictions on data availability

The data that support the findings of this study are available from the corresponding author upon request. The high-throughput sequencing data from this study have been deposited in the NCBI BioProject database under the accession codes PRJNA727868 and PRJNA727869. Supplementary information is available in the online version of the paper.

Field-specific reporting

Please select the one below that is the best fit for your research. If you are not sure, read the appropriate sections before making your selection.

☒ Life sciences ☐ Behavioural & social sciences ☐ Ecological, evolutionary & environmental sciences

For a reference copy of the document with all sections, see nature.com/documents/nr-reporting-summary-flat.pdf

Life sciences study design

All studies must disclose on these points even when the disclosure is negative.

Sample size	No statistical methods were used to predetermine sample size for experiment. Sample size of protoplasts was chosen through the nylon mesh (0.1mm). The experiments of protoplasts transfection were performed with three biological repeats. About 500,000 lettuce protoplasts and 250,000 rapeseed protoplasts were used for transfection. The number of protoplasts was measured by hemacytometer. All calli and shoots were sampled and analyzed the mutation by Sanger sequencing and targeted deep sequencing.
Data exclusions	No data was excluded.
Replication	All attempts for replication were successful. Experiments were done in three independent replicates.
Randomization	Lettuce and rapeseed protoplasts were isolated and randomly separated for each transfection.
Blinding	Samples were blindly processed without description during DNA extraction, targeted deep sequencing and Sanger sequencing by different researchers. In some cases automated procedures (targeted deep sequencing and Sanger sequencing) that should not bias outcomes.

Reporting for specific materials, systems and methods

We require information from authors about some types of materials, experimental systems and methods used in many studies. Here, indicate whether each material, system or method listed is relevant to your study. If you are not sure if a list item applies to your research, read the appropriate section before selecting a response.

Materials & experimental systems

n/a	Involved in the study	
☒	☐	Antibodies
☒	☐	Eukaryotic cell lines
☒	☐	Palaeontology and archaeology
☒	☐	Animals and other organisms
☒	☐	Human research participants
☒	☐	Clinical data
☒	☐	Dual use research of concern

Methods

n/a	Involved in the study	
☒	☐	ChIP-seq
☒	☐	Flow cytometry
☒	☐	MRI-based neuroimaging
