# Supplementary figures and images for: Chloroplast and mitochondrial DNA editing in plants
Source: Nat Plants. 2021 Jul 1;7(7):899–905. doi: 10.1038/s41477-021-00943-9 (PMC8289734; doi:10.1038/s41477-021-00943-9)

## Unprocessed gels for Supplementary Figure 7

PCR

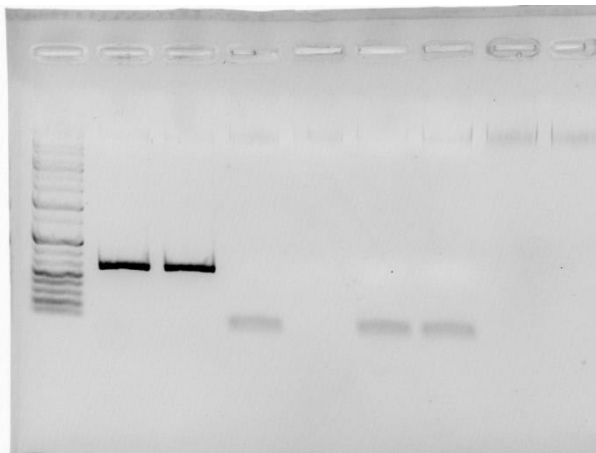

RT-PCR

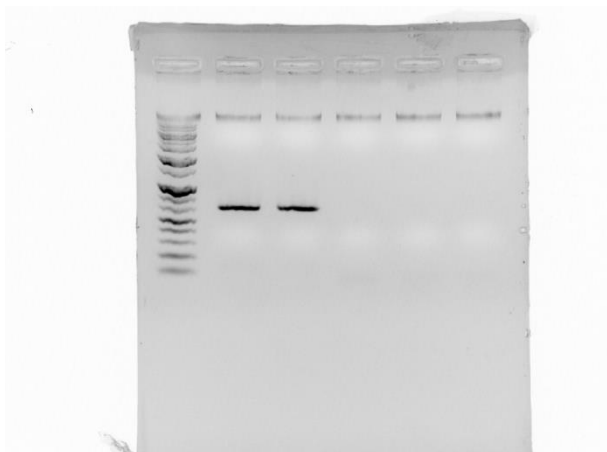

Supplement: Supplementary file 3 — Unprocessed gels for Supplementary Fig. 7. [file 41477_2021_943_MOESM3_ESM.pdf]
